# Inequalities of caries experience in Nevada youth expressed by DMFT index vs. Significant Caries Index (SiC) over time

**DOI:** 10.1186/1472-6831-11-12

**Published:** 2011-04-05

**Authors:** Marcia Ditmyer, Georgia Dounis, Connie Mobley, Eli Schwarz

**Affiliations:** 1 UNLV School of Dental Medicine, 1001 Shadow Lane, MS 7425, Las Vegas, NV 89106, USA; 2 University of Sydney, Faculty of Dentistry, Population Oral Health, USA; 3 Oregon Health & Science University, School of Dentistry, Portland, OR, USA

## Abstract

**Background:**

With the increasingly polarized distribution of dental caries among children and adolescents, the usual DMFT measure has become a less meaningful population descriptor. To re-focus on identifying the high caries prevalence group the Significant Caries Index (SiC) was created. The aims of this study were to analyze the prevalence and severity of dental caries in Nevada youth over a period of eight years and to compare its expression by means of DMFT and SiC; analyze the caries trends in the population and their underlying factors, and determine whether Nevada youth were at risk for significantly high levels of dental caries.

**Methods:**

Retrospective data was analyzed from a series of sequential, standardized oral health surveys across eight years (2001/2002-2008/2009) that included over 62,000 examinations of adolescents 13-19 years of age, attending public/private Nevada schools. Mean Decayed-Missing-Filled Teeth index (DMFT) and Significant Caries Index (SiC) were subsequently computed for each academic year. Descriptive statistics were reported for analysis of comparative DMFT and SiC scores in relation to age, gender, racial background, and residence in a fluoridated/non-fluoridated community. Logistic regression analysis was used to analyze the differential impact of the variables on the probability of being in the high caries prevalence group.

**Results:**

Comparison of students' mean DMFT to National (NHANES) data confirmed that dental caries remains a common chronic disease among Nevada youth, presenting higher prevalence rates and greater mean scores than the national averages. Downward trends were found across all demographics compared between survey years 1 and 6 with the exception of survey year 3. An upward trend began in survey year six. Over time, the younger group displayed an increasing proportion of cariesfree individuals while a decreasing proportion was found among older examinees. As expected, the mean SiC score was significantly higher than DMFT scores within each survey year across comparison groups (p < 0.001).

**Conclusions:**

Using both caries indices together may help to highlight oral health inequalities more accurately among different population groups within the community in order to identify the need for special preventive oral health interventions in adolescent Nevadans. At the community level, action should focus on retaining and expanding the community fluoridation program as an effective preventive measure. At the individual level the study identifies the need for more targeted efforts to reach children early with a focus on females, Hispanics and Blacks, and uninsured children.

## Background

For many years, the World Health Organization (WHO) global goal for year 2000 for dental caries of no more than an average of 3 DMFT (decayed, missing, filled teeth) at 12 years of age has been used as a global yardstick for oral health program success [1]. Decades ago the WHO developed oral disease surveillance systems to monitor dental caries in children. The first global map with DMFT data on 12-year-olds was presented in 1969. This map indicated high prevalence of caries in industrialized countries and generally low values in the developing countries [1]. Although dental caries prevalence in industrialized countries has declined significantly since the early 1970s, oral diseases, including caries, remain a major public health challenge [2-4]. In 2007 the WHO reported that 60-90% of school children worldwide have dental caries [5]. Traditional dental care remains a significant economic burden for many countries, where 5-10% of public health expenditure relates to oral health [5]. In US children, the recently reported prevalence of dental caries was approximately 60% in ages 12 to 19, with a reported 20% having untreated tooth decay [4]. Childhood dental caries has been reported to be the most prevalent infectious disease in our nation - 5 times as common as asthma and 7 times as common as hay fever [6]. A review of progress towards meeting the *Healthy People 2010 *Objectives for Oral Health, noted that 11 of the objectives have shown little or no progress [6]. In 2004, most American children reported good oral health, but subsets suffered a higher level of oral disease, primarily children living in poverty and some racial/ethnic minority populations [7]. Seventy-eight (78) percent of 17-year-olds had experienced tooth decay. One in four American children was born into poverty, suffering twice as much tooth decay as their more affluent peers and more likely having no access to oral health care [7]. Children from families without medical insurance were 2.5 times less likely than insured children to receive dental care and 3 times more likely than insured children to have unmet dental needs [7]. The use of DMFT scores to establish severity and prevalence of caries is an accepted practice in the dental community and has continuously been used to assess prevalence in the National Health and Nutrition Examination Survey (NHANES) [4]. An analysis conducted by the WHO found that there was a skewed distribution of caries prevalence in many countries; a significant proportion of 12-year-olds still had high or even very high DMFT values even though a proportion was totally cariesfree [8]. This polarization of the caries picture has the effect of making the mean DMFT value less meaningful as a population descriptor in that it does not accurately reflect the burden of disease [9]. Thus it may lead to the incorrect conclusion that the caries situation for the whole population is under control, whereas in reality population subgroups still suffer from high caries rates. The Significant Caries Index (SiC) was introduced in 2000 to bring attention to the individuals with the highest caries values in each population [8,10-12]. The aims of this study were to analyze the prevalence and severity of dental caries in Nevada youth over a period of eight years and to compare its expression by means of DMFT and SiC; analyze the caries trends in the population and their underlying factors; and determine whether Nevada youth were at risk for significantly high levels of dental caries and should be specifically targeted for oral health interventions.

## Methods

### Selection and description of study population

Since 2001, we have been conducting a grant-sponsored, annual statewide, school-based, oral health screening initiative in public/private middle and high schools in Nevada. These sequential cross-sectional studies were conducted from 2001/2002 academic year through the 2008/2009 academic year and comprised 62,707 oral health examinations of adolescents between ages 13 and 19. Inclusion criteria for participation were parental consent and student assent. The University of Nevada Las Vegas Institutional Review Board approved these surveys which also ensured student confidentiality and protection.

### Oral Health Screening

Examinations were conducted in dedicated mobile dental clinics (one each in northern and southern Nevada). All licensed professional serving as examiners for the study were calibrated at the start of each school year by the grant administrator in accordance with the procedural manual established for and approved by the grant funding agency and the university institutional review board. Trained and calibrated licensed oral health professionals served as examiners and performed oral health screenings to assess DMFT scores. Single measure intraclass correlation was assessed as an index of the reliability of a single examiner (r = 0.81) and interrater reliability between all examiners were computed with the average measure intraclass correlation coefficient (ICC) (r = 0.98) [13].

Examiners followed the Radike criteria with modifications to establish prevalence (untreated and restored lesions and untreated dental caries) [14]. Artificial light and non-magnifying mirrors were used to perform visual assessments similar to methods used in NHANES [4]. Unlike NHANES, restrictions placed by the funding agency disallowed the use of compressed air and explorers. However, when comparing studies using visual methods without probing and drying, to studies using visual/tactile methods with explorers and compressed air, only in groups with low caries prevalence were statistical differences observed [15]. As with NHANES, severity was determined using DMFT indices developed by Klein et al. [16]. The oral screening initiative procedural manual detailed all diagnostic and coding criteria.

### Face-to-Face Interviews

Trained interviewers collected demographic and oral health status information through face-to-face interviews in the privacy of the mobile clinic setting. Selected self-reported information identified behaviors, health history, and environmental factors of interest. Cronbach's alpha was used to assess internal reliability of the questionnaire (r = 0.79) [17].

### Statistical Analysis

DMFT describes the severity of dental caries in an individual. It is calculated by adding the number of decayed (D), missing (M), and Filled (F) teeth and expresses the individual's dental caries experience until the day of examination. Because the four 3rd molars erupt at approximately age 17 (average between ages 15-25) these teeth were excluded from the DMFT calculation in this population. The sum of all the DMFT values divided by the total number of individuals in the sample provides the mean DMFT for the population [7,18].

The Significant Caries Index (SiC) was calculated as follows [8]:

1) Individuals in the population (sample) were sorted according to their DMFT values

2) One third of the population with the highest caries scores was selected

3) The mean DMFT for this subgroup was calculated. This value is the SiC Index.

DMFT and SiC values were computed for each survey year and were further analyzed by demographic variables to include age, gender, race/ethnicity, whether they resided in areas where the municipal water supply was fluoridated, and whether or not they had dental insurance. Descriptive statistics (mean and standard error) were used to compare and compute DMFT and SiC index differences between the subgroups. T-tests and ANOVAs were computed between DMFT Scores and SiC Indices. In order to analyze the relative risk of having a high caries score in relation to our selected demographic variables, a bivariate logistic regression analysis was carried out for each year; with the population dichotomized according to individuals in the SiC group (group with the highest DMFT, coded as 1) and all others (coded as 0). Wald statistics was used to determine significance of the respective variables. Tests were done to determine the presence of multicollinearity (none found). Backwards stepwise method was used; Odds ratios (OR) produced and data reported in this study were analyzed using SPSS 18.0 (SPSS, Inc., Chicago, IL).

## Results

Demographics of those screened are shown in Table 1. During the eight years of survey the annually examined populations varied between 6,400 and 10,900 individuals. Apart from the first survey year the younger group (13-15 years old) stabilized at around 2/3 of the ones examined, whereas the older group (16-19 years old) comprised around 1/3. Throughout the surveys the gender balance was almost 50:50. There was a downward trend in the proportion of adolescents of White background, whereas both Blacks and Hispanics showed increasing proportions of the examined populations. The relative distribution of the study groups according to residence varied throughout the period with Clark County residents consistently underrepresented (Clark County comprises around 72% of Nevada's population). During the first 4 years of survey insurance status was relatively stable across the entire period with around 2/3 reporting insured status and 1/3 not insured.

**Table 1:** Study population decription

Variable	Year 1		Year 2		Year 3		Year 4		Year 5		Year 6		Year 7		Year 8	
	2001-2002		2002-2003		2003-2004		2004-2005		2005-2006		2006-2007		2007-2008		2008-2009	
	**N-value**	**%**	**N-value**	**%**	**N-value**	**%**	**N-value**	**%**	**N-value**	**%**	**N-value**	**%**	**N-value**	**%**	**N-value**	**%**
**Total**	9471	100.0	7488	100.0	10,915	100.0	6590	100.0	8438	100.0	6568	100.0	6868	100.0	6379	100.0
**Age**																
13-15	4880	51.5	5184	69.2	7428	68.1	4850	73.6	5971	70.8	4662	70.9	4684	68.2	4681	73.4
16-19	4591	49.5	2304	30.8	3487	31.9	1740	26.4	2467	29.2	1906	29.1	2184	31.8	1698	26.6
**Sex**																
Male	4710	49,7	3588	47.9	5370	49.2	3047	46.2	4132	49.0	3169	48.2	3329	48.5	3166	49.6
Female	4761	50.3	3900	52.1	5539	50.8	3543	53.8	4306	51.0	3385	51.6	3539	51.5	3213	50.4
**Race**																
White, NH	6825	72.1	4755	63.5	6322	57.9	3699	56.1	4187	49.6	3663	55.8	3731	54.3	3454	54.1
Black, NH	658	6.9	665	8.9	1032	9.5	621	9.4	763	9.0	552	8.4	556	8.1	668	10.5
Hispanic	1988	21.0	2068	27.6	3561	32.6	3063	46.5	3488	41.4	2353	35.8	2581	37.6	2257	35.4
**Fluoridation**																
Clark County	3218	33.9	2121	28.3	5472	50.1	1670	25.4	4290	50.8	2762	42.1	2985	43.5	3042	47.7
All others	6253	66.1	5367	71.7	5443	49.9	4918	74.6	4148	49.2	3806	57.9	3883	56.5	3337	52.3
**Insurance**																
Insured	N/A		5049	67.4	7357	67.4	4322	65.6	5743	68.1	4554	69.4	4454	64.8	3897	61.1
Non-insured	N/A		2439	32.6	3558	32.6	2268	34.4	2695	31.9	2004	30.6	2414	35.2	2482	38.9

Demographic breakdown of selected variables of study population of Nevada adolescents by year of survey.

The mean DMFT values by year is detailed in Table 2. Overall, the DMFT index stayed around 3 DMFT at the beginning and at the end of the survey period. However, during the period some decrease took place, which reverted to an increase towards the end of the survey years. This trend was reflected for the two age groups as well with the younger age group at a significant lower level than the older. Both age groups had significantly higher DMFT score than the comparable national samples of the same age illustrated by the NHANES figures in the table. Females had consistently higher DMFT than males and Hispanic examinees were consistently higher than Whites and Blacks. Residents of the fluoridated Clark County were consistently lower than residents of the rest of the State, and insured residents exhibited less caries experience than the non-insured group.

**Table 2:** Caries severity among Nevada adolescents

Variable	Year 1	Year 2	Year 3	Year 4	Year 5	Year 6	Year 7	Year 8	NHANES
	2001-2002	2002-2003	2003-2004	2004-2005	2005-2006	2006-2007	2007-2008	2008-2009	1999-2004
	N = 9471	N = 7488	N = 10,915	N = 6590	N = 8438	N = 6558	N = 6868	N = 6379	NA
	**M (SE)**	**M (SE)**	**M (SE)**	**M (SE)**	**M (SE)**	**M (SE)**	**M (SE)**	**M (SE)**	
**Total**	3.06 (0.04)	2.94 (0.04)	3.13 (0.03)	2.59 (0.04)	2.77 (0.03)	2.52 (0.04)	2.92 (0.05)	3.06 (0.04)	
**Age**									
13-15	3.73 (0.06)	2.66 (0.04)	2.80 (0.04)	2.40 (0.04)	2.44 (0.04)	2.24 (0.05)	2.50 (0.05)	2.71 (0.05)	1.78 (0.08)
16-19	4.36 (0.06)	3.58 (0.08)	3.82 (0.07)	3.15 (0.09)	3.58 (0.07)	3.20 (0.09)	3.80 (0.08)	4.04 (0.09)	3.31 (0.09)
**Sex**									
Male	3.87 (0.06)	2.76 (0.05)	2.94 (0.05)	2.42 (0.06)	2.65 (0.05)	2.42 (0.06)	2.79 (0.06)	2.82 (0.06)	2.31 (0.09)
Female	4.20 (0.06)	3.11 (0.05)	3.31 (0.05)	2.73 (0.06)	2.89 (0.05)	2.62 (0.06)	3.03 (0.06)	3.30 (0.06)	2.79 (0.08)
**Race**									
White, NH	3.05 (0.05)	2.65 (0.05)	2.86 (0.05)	2.32 (0.05)	2.45 (0.05)	2.26 (0.05)	2.61 (0.05)	2.63 (0.05)	2.54 (0.10)
Black, NH	3.77 (0.14)	3.35 (0.13)	3.40 (0.09)	2.82 (0.14)	2.92 (0.12)	2.75 (0.14)	2.86 (0.15)	3.39 (0.13)	2.20 (0.10)
Hispanic	4.08 (0.09)	3.47 (0.08)	3.52 (0.06)	2.99 (0.07)	3.13 (0.06)	3.69 (0.06)	3.37 (0.07)	3.63 (0.07)	2.82 (0.13)
**Fluoridation**									
Clark County	2.62 (0.06)	2.72 (0.07)	3.05 (0.04)	2.65 (0.07)	2.50 (0.04)	2.40 (0.06)	2.35 (0.05)	2.62 (0.06)	
All others	3.47 (0.05)	2.91 (0.06)	3.22 (0.07)	2.59 (0.05)	2.06 (0.05)	2.61 (0.06)	3.35 (0.06)	3.47 (0.05)	
**Insurance**									
Insured	N/A	2.93 (0.04)	3.10 (0.04)	2.59 (0.05)	2.72 (0.04)	2.61 (0.05)	2.68 (0.05)	2.83 (0.05)	
Non-insured	N/A	3.29 (0.09)	3.41 (0.09)	2.95 (0.10)	3.25 (0.09)	2.94 (0.09)	3.64 (0.11)	3.83 (0.12)	

Number of Decayed, Missing, and Filled Teeth (mean DMFT and standard error) of Nevada adolescents by year of study and in relation to selected variables.

Note. M = mean DMFT Score; SE = standard error

**Figure 1:**
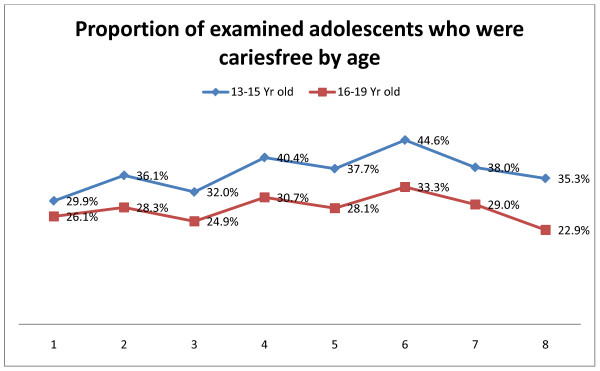
**Cariesfree adolescents by age**. Proportion of examined adolescents in each year who were cariesfree in relation to age group. Numbers 1-8 indicates year of examination from 2001/2002 - 2008/2009.

In order to assess the polarization in the caries picture over time, Figure 1 illustrates the proportion of the examinees who were cariesfree. Overall, in year 1 28% were cariesfree and around 32% were cariesfree in year 8. But it was evident that the younger and the older groups diverged over time with an increase in the cariesfree younger persons from around 30% to around 35% and a decrease among the older persons, from 26% to 22%. Conversely, this figure also illustrates that the prevalence of dental caries in the younger group was around 65% and around 77% in the older group.

**Figure 2:**
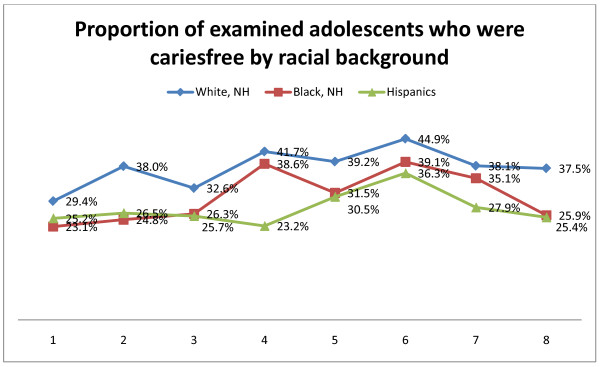
**Cariesfree adolescents by racial background**. Proportion of examined adolescents in each year who were cariesfree in relation to racial background. Numbers 1-8 indicates year of examination from 2001/2002 - 2008/2009.

**Figure 3:**
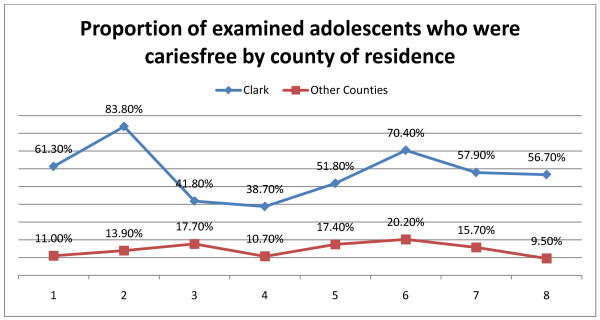
**Cariesfree adolescents by residence**. Proportion of examined adolescents in each year who were cariesfree in relation to their county of residence. Clark County is fluoridated, all other counties are non-fluoridated. Numbers 1-8 indicates year of examination from 2001/2002 - 2008/2009.

The variation of cariesfree individuals over time in relation to racial background and county of residence respectively are illustrated in Figure 2 and Figure 3. It is interesting to note that the white population reflected a general increase in proportion of cariesfree individuals (from around 29% to around 38%), whereas the two other groups started out around 25%, improved somewhat during the survey years, but ended up around 25-26% in year 8 (Figure 2). According to county of residence a dramatic difference was evident in that the proportion of cariesfree individuals in fully fluoridated Clark County varied around 40-80%, whereas only around 10-20% in all other counties (not fluoridated) were found to be cariesfree.

The SiC indices by survey year is detailed in Table 3. It comprises the 30% of the examined population with the highest DMF values and thus, the mean caries values were considerably higher than the corresponding DMF values ranging from an initial 8.86 DMF to 6.01 in the middle of the period and then climbing up to 7.0 DMFT.

**Table 3:** Caries severity among Nevada adolescents with high caries scores

Variable	Year 1	Year 2	Year 3	Year 4	Year 5	Year 6	Year 7	Year 8
	2001-2002	2002-2003	2003-2004	2004-2005	2005-2006	2006-2007	2007-2008	2008-2009
	N = 3157	N = 2496	N = 3638	N = 2197	N = 2812	N = 2189	N = 2289	N = 2126
	**M (SE)**	**M (SE)**	**M (SE)**	**M (SE)**	**M (SE)**	**M (SE)**	**M (SE)**	**M (SE)**
**Total**	8.86 (.005)	6.85 (0.56)	6.85 (0.06)	6.18 (0.06)	6.01 (0.05)	6.31 (0.07)	6.84 (0.06)	7.00 (0.06)
**Age**								
13-15	8.93 (0.08)	6.56 (0.06)	6.56 (0.06)	5.89 (0.06)	5.65 (0.06)	6.08 (0.08)	6.46 (0.07)	6.74 (0.07)
16-19	8.81 (0.07)	7.36 (0.10)	7.36 (0.10)	6.96 (0.12)	6.66 (0.09)	6.70 (0.12)	7.40 (0.09)	7.45 (0.10)
**Sex**								
Male	8.77 (0.08)	6.70 (0.05)	6.70 (0.08)	6.08 (0.09)	5.99 (0.07)	6.12 (0.09)	6.74 (0.08)	6.92 (0.09)
Female	8.93 (0.08)	6.97 (0.05)	6.97 (0.08)	6.26 (0.08)	6.03 (0.06)	6.48 (0.09)	6.92 (0.8)	7.06 (0.08)
**Race**								
White, NH	8.97 (0.07)	6.77 (0.08)	6.77 (0.08)	6.02 (0.08)	5.72 (0.07)	5.92 (0.09)	6.64 (0.08)	6.68 (0.09)
Black, NH	8.61 (0.21)	6.95 (0.16)	6.78 (0.15)	6.47 (0.19)	5.79 (0.15)	6.19 (0.22)	6.67 (0.21)	6.86 (0.11)
Hispanic	8.57 (0.11)	7.08 (0.09)	7.08 (0.09)	6.31 (0.09)	6.39 (0.07)	6.91 (0.11)	7.12 (0.09)	7.27 (0.09)
**Fluoridation**								
Clark County	8.19 (0.07)	6.81 (0.14)	6.56 (0.06)	6.22 (0.11)	5.54 (0.06)	6.09 (0.09)	6.29 (0.09)	6.44 (0.08)
All others	8.99 (0.09)	6.96 (0.14)	7.36 (0.10)	7.17 (0.07)	6.46 (0.07)	7.45 (0.09)	7.14 (0.07)	7.39 (0.08)
**Insurance**								
Insured	N/A	6.90 (0.07)	6.90 (0.07)	6.16 (0.07)	6.00 (0.06)	6.39 (0.08)	6.71 (0.07)	6.96 (0.08)
Non-insured	N/A	7.04 (0.12)	7.35 (0.11)	6.40 (0.16)	6.43 (0.13)	7.19 (0.15)	7.17 (0.14)	7.24 (0.13)

Number of Decayed, Missing, and Filled Teeth (mean DMFT and standard error) among the 30% subgroup of Nevada adolescents with the highest DMFT scores (Significant caries Index group) by year of study and in relation to selected variables.

Note. M = mean DMFT Score; SE = standard error

The SiC Index was significantly higher than mean DMFT Index when comparing each demographic variable within each survey year (p < 0.001). Females, older adolescents (16-19 year olds), minority groups, those living in areas where the municipal water supply is not fluoridated, and those without dental insurance had higher mean DMFT scores. SiC Indices were significantly higher across the board, with the results paralleling the trends in the mean DMFT scores

In order to assess the relative impact of the independent demographic variables on the probability of ending up in the high caries group we performed a bivariate logistic regression analysis. The resulting Wald Statistics as shown in Table 4 indicated that each of the independent variables was highly statistically significant. The pattern of the logistic regression analysis changed little from one year to the next. The probability of being in the SiC group, i.e. being among the 30% of individuals with the highest DMFT scores was related to being older (OR between 1.6-1.9), being female (OR around 2.0), being Hispanic (OR around 2.0) or Black (OR between 1.4-1.8), residing in a non-fluoridated community (OR between 1.8-2.8) or being non-insured (OR between 2.0 - 2.4)(Table 5).

**Table 4:** Wald statistics outcome after logistic regression analysis

Variable	Year 1	Year 2	Year 3	Year 4	Year 5	Year 6	Year 7	Year 8
**Age**	20.73*	49.50**	62.50**	28.40**	68.90**	87.81**	91.90**	84.10**
**Sex**	16.96*	14.83*	24.20**	12.90*	22.59**	12.52*	14.13*	28.40**
**Race**	18.81*	98.40**	45.56**	40.60**	62.40**	48.78**	60.12**	75.14**
**Fluoridation**	45.08**	14.64*	14.80*	19.80*	16.20*	12.87*	23.10**	19.81*
**Insurance**	N/A	18.50*	17.96*	11.50*	19.40*	12.50*	29.68**	19.20*

Statistical significance of individual independent variables expressed as the Wald Statistic as an outcome of the logistic regression analysis by year of survey.

Note. * P < 0.05; ** p < 0.001

**Table 5:** Caries severity among Nevada adolescents

Variable	Year 1 OR (CI)	Year 2 OR (CI)	Year 3 OR (CI)	Year 4 OR (CI)	Year 5 OR (CI)	Year 6 OR (CI)	Year 7 OR (CI)	Year 8 OR (CI)
**Age**								
13-15	*	*	*	*	*	*	*	*
16-19	1.95 (1.79-2.05)	1.68 (1.45-1.75)	1.66(1.62-1.89)	1.641.22-1.82)	1.64(1.57-1.71)	1.59(1.53-1.66)	1.87(1.42-2.05)	1.68(1.44-1.79)
**Sex**								
Male	*	*	*	*	*	*	*	*
Female	2.04(1.78-2.35)	1.86(1.76-1.95)	1.85(1.75-1.89)	1.98(1.75-2.24)	1.92(1.76-2.15)	2.23(1.88-2.57)	2.43(1.67-2.84)	1.98(1.56-2.34)
**Race**								
White, NH	*	*	*	*	*	*	*	*
Black, NH	1.75(1.67-1.85)	1.82(1.77-1.97)	1.75(1.49-1.97)	1.74(1.62-1.76)	1.68(1.60-1.75)	1.66(1.59-1.74)	1.45(1.29-1.70)	1.59(1.42-1.78)
Hispanic	1.96(1.67-2.03)	2.34(1.82-2.68)	1.96(1.82-1.13)	2.05(1.75-2.58)	1.96(1.82-1.13)	1.98(1.73-2.33)	1.87(1.65-2.05)	2.33(1.82-2.87)
**Fluoridation**								
Clark County	*	*	*	*	*	*	*	*
All others	2.76(1.76-2.85)	1.86(1.67-2.04)	1.76(1.67-1.89)	2.13(1.93-2.43)	1.92(1.88-2.11)	1.97(1.49-2.17)	2.04(1.75-2.17)	2.05(1.68-2.57)
**Insurance**								
Insured	N/A	*	*	*	*	*	*	*
Non-insured	N/A	1.97 (1.82-1.34)	2.12(1.96-2.37)	1.96(1.82-2.15)	2.02(1.87-2.14)	1.99(1.28-2.34)	2.38(1.80-2.87)	2.01(1.69-2.77)

Odds Ratios (Confidence Interval) of being in the SiC group by demographic variable by year of survey

Note. * = Reference group; OR = Odds Ratio; CI = 95% Confidence Interval

## Discussion

This study investigated the caries prevalence and severity trends in Nevada youth using both the mean DMFT index and the SiC Index. The use of DMFT has been an accepted practice for assessing the prevalence and severity of caries in a population [4]. However the epidemiologic changes in the dental caries picture during the last 2-3 decades, have made it increasingly evident that mean DMFT values do not capture the polarized caries development with a more skewed distribution of caries [8]. Mean DMFT values are an average of all members of the population, irrespective of the distribution of the severity of the disease within the population.

### Mean DMFT Trends

Eight years of oral health screening data were collected through the Oral Health Surveillance Initiative across the state of Nevada, with over 62,000 adolescents participating. The mean DMFT from these data, compared to national NHANES data confirmed that dental caries remains a common chronic disease among Nevada youth, and that Nevada youth present with higher prevalence rates and greater mean DMFT indices than the national average (Table 2) [4]. Where the NHANES study reported a prevalence of around 51% among the younger group and around 67% in the older group [4] we found 65% and 77% respectively (Figure 2). Furthermore, because this sample was assessed using a modified protocol, data from this study may be an underestimate of caries prevalence compared to NHANES data. Improvements were found in mean DMFT scores across all demographics compared (age, sex, race group, whether residing in fluoridated area, and dental insurance status) between year 1 and year 6 with the exception ofyear 3. However since year six, there has been a trend towards more caries and less cariesfree individuals in all demographics (Figures 1, 2, 3). Although dental caries is largely preventable, it remains the most common chronic disease of children aged 6 to 11 years (25%) and adolescents aged 12 to 19 years (59%) [4,18,19]. Additionally, certain segments of the population (e.g., members of racial or ethnic minority groups, sex, and older children) have more dental decay, much of which remains untreated [18,20,21].

### SiC Index Trends

The same eight year data set was used to compute the SiC Index. As expected, the SiC Index was significantly higher in all comparisons with DMFT (p < 0.001). A comparison between the two indices, indicated there is a large Nevada youth population subgroup that presented with a significantly higher caries rate than the targeted mean DMFT score of 3.0 [1]. The Mean DMFT score for Nevada youth, although higher than the national average and in some of the subgroups higher than the targeted 3.0 mean DMFT, demonstrates how skewed the present caries problem is and does not reflect the true extent of caries prevalence or severity in all subgroups of the population. In fact, the mean of the highest caries scorers is close to three times the population mean. Recently, Sheiham et al. [22] have pointed out that caries patterns seem to exist in subgroups of a population with those groups remaining at a stable relative position to each other even when prevalence changes over time. This pattern seems to be reflected in this study. It is of note that the present population is in fact 8 populations of the same age studied over eight years sequentially. In no instance is any specific sub-group found to be veering away from its pattern of dental caries or crosses into the pattern of another group. For instance, the younger group remains at the lower level of dental caries and even improves through increasing rates of freedom of caries. With regard to racial background, the white examinees remain at the lowest caries level of the racial groups.

The use of the SiC Index that includes DMFT can elucidate interpretations of findings, especially in situations where resources are limited for interventions. While mean scores provide a good measure of population disease levels, it is important to also look at those who might be carrying a significant burden of the dental disease experience in the population. To help in identifying high risk groups, it is recommended to calculate the SiC Index at several levels. It has been suggested that a SiC goal be established so that public health professionals can have 2 goals: mean DMFT and SiC Index. One [10] suggested a SiC of less than 3.0 in 12-year-old children as a global oral health goal to be achieved by the year 2015, while another suggested that a SiC global goal of less than 5.0 be set for 15-year olds rather than the WHO targeted age of 12-year olds [23].

### Interpretation of Trends

Downward trends were found in both Mean DMFT scores and SiC Indices across all demographics compared (age, sex, race group, whether residing in fluoridated area, and dental insurance status) between year 1 and year 6 with the exception of year 3. Althought these trends were parallel, the SiC Indicies were significantly higher than the mean DMFT Index across all years. Since year six, there has been an upward trend in all demographics (Figures 1, 2, 3). Data showed that minority children had higher prevalence of caries. Research demonstrates that minority children are more likely to experience tooth decay and have their cavities untreated [18]. Because children of color are the fastest growing subpopulation of children in the U.S., their higher caries experience predicts an upturn in disease prevalence over the coming years unless special efforts are made to address their oral health needs [18].

Community water fluoridation has been ranked as one of the ten great public health achievements of the 20th century [18,24,25]. *Healthy People 2010 *objectives seek to eliminate health disparities ensuring that all Americans receive the benefits of good oral health. Community-based programs, such as community water fluoridation are cost-effective ways to achieve this goal [24,25]. This study found that those children living in communities with fluoridated municipal water supplies experience substantially lower mean DMFT scores. This has special importance in Nevada where attempts to expand the fluoridation program to counties other than Clark County have met with considerable resistance.

Inadequate access to dental care for children of low-income families may be largely due to lack of dental insurance [26]. In one study, subjects without dental insurance were 20-40% more likely to present with higher mean DMFT indices than those with insurance [26]. Despite improvements in children's oral health through prevention, dental caries remained the most common chronic childhood disease in the US during the twentieth century [26].

A report released by the CDC [18] in 2002 reported a 15.2% increase in disease among the nation's *youngest *children ages 2 through 5 years. Because tooth decay in the primary teeth predicts future tooth decay in permanent teeth, the upturn in caries experience in preschoolers could be expected to continue in permanent teeth. A recent epidemiologic review found that in many countries there is a marked increase in dental caries prevalence that affects both children and adults [27]. This could partially explain the upward trend in caries prevalence observed in this study from early 2000. Overall this study found that older adolescents, those of racial groups, those who live in non-fluroidated areas, and those without dental insurance all experienced higher mean DMFT scores in all years included in this report. The trend line for each of the comparison groups was similar (Figures 2, 3, 4). With reference to the previous part of the discussion we would expect that the relative stability of the subgroups in a special caries pattern could be used to conduct an early identification of the children with the highest caries prevalence and incidence, because they would be considered a special risk group for ending up in late adolescence with considerably higher caries rates than the remainder of the population.

### Limitations and Future Recommendations

Self-reports warrant some caution in interpreting those data. However, data collection and entry protocols were well documented and quality control guidelines were implemented during the oral health screening process throughout the time span reported. Due to confidentiality issues, students could not be tracked over time preventing longitudinal data collection, therefore to help strengthen the data; analysis of cross sectional data across all years by school was examined to reduce the likelihood of repeat students. Since the sample was so large, assumptions could be made that all would have access to similar secondary fluoride influence, for example all would have access to fluoride toothpaste, as well as water that contains fluoride. Inclusion of these other potential sources of fluoride supplementation may influence these results. While this study focused on comparisons of results using traditional calculations of mean DMFT and the Significant Caries Index, it doesn't make comparisons between those who are cariesfree and those with the highest reported mean DMFT. Future reports may examine these differences for more in-depth interpretations.

## Conclusions

The series of sequentially conducted epidemiologic studies demonstrated that downward trends were found in both mean DMFT scores and SiC Indices across all demographics compared (age, sex, race group, whether residing in fluoridated area, and dental insurance status) between year 1 and year 6. Since year six, there has been an upward trend in all demographics. An increasing divergence in caries prevalence was found between younger and older adolescents and between individuals of white and all other racial backgrounds. The mean DMFT values did not accurately reflect the skewed distribution of dental caries in Nevada youth leading to incorrect conclusions that the caries rate for the statewide population is under control. The data indicate there is a large proportion of adolescents in Nevada with poor oral health status, which only seems to improve very slowly. Decreasing caries prevalence in all countries is important to promote overall general health. The probability of ending up in the high caries group was consistently related to being older, being female, being Hispanic or Black, residing in a non-fluoridated community, and not having insurance cover.

Findings from this study should aid in two ways. At the community level, the study strongly points to the importance of retaining and expanding the community fluoridation program as an effective preventive measure. At the individual level the study identifies the need for more targeted efforts to reach children early with a focus on females, Hispanics and Blacks, and uninsured children.

## Competing interests

The authors declare that they have no competing interests.

## Authors' contributions

MD, GD, and CM have been engaged in the multi-year development of the surveillance program, including developing the protocols and guidance for examiners, data collection as well as ongoing quality control. MD performed the statistical analysis in collaboration with ES. The paper was drafted by MD, ES, ED, and CM contributed to its completion. All authors have read and approved the final manuscript.

## Pre-publication history

The pre-publication history for this paper can be accessed here:

http://www.biomedcentral.com/1472-6831/11/12/prepub
